# Association of maternal serum vitamin a levels in the first trimester with the risk of adverse pregnancy outcomes: a prospective cohort study of Chinese women

**DOI:** 10.3389/fnut.2026.1735875

**Published:** 2026-04-02

**Authors:** Hui Yuan, Yang Wang, Qinqin Ren, Xingjiang Ji, Jie Yang, Yijin Wang, Yao Liu, Yuanhuan Wei, Ruifang Sun, Hongguang Yang, Ping Tian, Jianjun Yang, Guifang Deng

**Affiliations:** 1 Department of Public Health, School of Public Health, Ningxia Medical University, Yinchuan, China; 2 Department of Clinical Nutrition, Shenzhen Nanshan People's Hospital, Shenzhen, Guangdong, China; 3 Department of Children Healthcare, Shenzhen Nanshan People's Hospital, Shenzhen, Guangdong, China

**Keywords:** adverse pregnancy outcomes, emergency caesarean section, gestational diabetes mellitus, low-birth-weight infant, small-for-gestational-age infant, vitamin A

## Abstract

**Purpose:**

To examine the association of serum vitamin A levels in early pregnancy with the risk of adverse pregnancy outcomes in Chinese women.

**Method:**

This prospective cohort study was conducted in the Department of Gynecology and Obstetrics at Shenzhen Nanshan People's Hospital from 2019 to 2020. Serum retinol concentrations were measured during the first trimester (the first 6–13 weeks) of pregnancy, and pregnancy outcomes were recorded in the hospital information system. Serum retinol concentrations were categorized into the following quintiles: Q1 ≤ 0.57 μmol/L, 0.57 < Q2 ≤ 0.63 μmol/L, 0.63 < Q3 ≤ 0.69 μmol/L, 0.69 < Q4 ≤ 0.82 μmol/L, and 0.82 < Q5 ≤ 2.50 μmol/L. The participants with serum retinol concentrations in the lowest quintile were used as the reference group. Statistical analysis was performed using multivariate logistic regression.

**Result:**

A total of 1,077 singleton pregnancies were included. After multivariable adjustment, serum retinol levels in the highest quintile (Q5) were associated with a significantly lower risk of gestational diabetes mellitus [odds ratio (OR): 0.50; 95% confidence interval (CI): 0.31–0.81]. Similarly, compared with the lowest quintile (Q1), the highest quintile (Q5) was associated with a reduced risk of low birth weight (OR: 0.26; 95% CI: 0.07–0.96). Moreover, the fourth quintile (Q4) was associated with a decreased risk of small for gestational age compared with Q1 (OR: 0.32; 95% CI: 0.12–0.82). In contrast, retinol levels in Q5 were associated with an increased risk of emergency cesarean section compared with Q1 (OR: 2.31; 95% CI: 1.26–4.26).

**Conclusion:**

Higher maternal serum retinol levels in the first trimester were associated with a reduced risk of gestational diabetes mellitus and low birth weight, but an increased risk of emergency cesarean section.

## Introduction

An adverse pregnancy outcome is an event that reduces the chance of having a healthy newborn (1). These outcomes encompass all the pathological complications that may occur during pregnancy and childbirth, such as pre-eclampsia, preterm birth, low birth weight (LBW), stillbirth, emergency cesarean section, and gestational diabetes mellitus (GDM). GDM is any degree of glucose intolerance with onset or first detection during pregnancy (2). It was reported that the global standardized total prevalence of GDM was 14.0% (3), being 14.8% in China (4). Adverse pregnancy outcomes are a major public health problem, as they increase maternal and neonatal mortality and affect the long-term health of both mothers and children (5). In addition, adverse pregnancy outcomes, particularly GDM, are associated with an increased risk of type 2 diabetes mellitus (T2DM) and cardiovascular disease in women after childbirth (4, 6–8), and with an increased risk of obesity and T2DM in their offspring (9). However, most adverse pregnancy outcomes can be diagnosed only in the mid-to-late stages of pregnancy, leaving insufficient time for prevention (10). Therefore, it is important to determine the risk factors for adverse pregnancy outcomes in early pregnancy.

Vitamin A is a fat-soluble compound that is essential for normal functioning of the human body. It can be obtained from the diet through consumption of meat containing retinol and its close derivatives, vegetables or dairy products containing provitamin A (carotenoids) or vitamin A-active carotenoids, fortified foods (e.g., cereals or juices), or vitamin supplements (11–13). During pregnancy, vitamin A is important for cell division, growth, and maturation of the fetal organs and skeletal system. It is also important for the development and maintenance of the fetal immune and visual systems (14, 15). However, high doses of vitamin A may exert teratogenic effects; such risks are predominantly reported in populations of developed countries (16). Women in these regions—excluding those with vitamin A deficiency—should avoid vitamin A supplementation and limit daily intake to no more than 700 micrograms (17). It is noteworthy that excessive vitamin A intake (e.g., >25,000 IU/day) may be associated with an increased risk of fetal urinary tract malformations. Therefore, it is generally recommended that daily intake should not exceed 10,000 IU before 60 days of gestation, and weekly intake should not exceed 25,000 IU, in order to reduce the risk of teratogenicity (18).

In addition to its effects on fetal development, vitamin A also possesses antioxidant properties, enabling it to directly scavenge reactive oxygen species (ROS) and protect cells from oxidative damage (19, 20). Fat-soluble vitamins such as vitamin A can block the initiation of free radical formation and inactivate free radicals (19–21). In the context of gestational diabetes mellitus (GDM), oxidative stress is believed to play a significant role in its pathogenesis. Oxidative stress, by interfering with proteins, lipids, and DNA, has been implicated in the pathogenesis of metabolic disorders of pregnancy including GDM (22, 23). An imbalance of oxidative stress in GDM is known to cause damage to vascular and pancreatic β-cells and can lead to adverse pregnancy outcomes (24, 25). Specifically, in women with GDM, the radical scavenger function is impaired alongside an overproduction of free radicals, resulting in elevated levels of oxidative stress markers (26, 27). Compared to normal pregnant women, pregnant women with type 2 diabetes mellitus (T2DM) exhibit reduced antioxidant capacity and elevated levels of oxidative products. Given the mechanistic similarities between GDM and T2DM, it is hypothesized that insufficient antioxidant defenses may contribute to the initiation and development of GDM (28–30). It has been observed that the activity of superoxide dismutase in pregnant women with GDM is significantly lower than that in normal pregnant women (18, 19). However, few studies have examined the relationship between serum vitamin A levels and GDM, and they have drawn inconsistent conclusions. For instance, Hekmat et al. (31) and Suhail et al. (32) surveyed significantly lower serum vitamin A levels in women with GDM during late pregnancy, while others have found no significant difference in serum vitamin A levels associated with GDM (33). Furthermore, retinol-binding protein 4 (RBP4), the primary transport protein for vitamin A, has been shown to be present at significantly higher concentrations in the serum of women with GDM (34–36). To date, only a limited number of prospective studies conducted in China have investigated the relationship between first-trimester serum vitamin A levels and the incidence of GDM. Research from Beijing, for instance, found that higher vitamin A levels in early pregnancy—even within the normal range—were positively associated with an increased risk of GDM (37, 38), a finding that is contrary to some initial hypotheses. Currently, several unresolved issues persist regarding the understanding of vitamin A during pregnancy. First, the exact association between vitamin A status and the risk of GDM remains unclear due to inconsistent findings from epidemiological studies. Second, there is a notable scarcity of prospective data specifically focusing on the first trimester, which represents a critical window for early pathophysiological changes. Therefore, clarifying the relationship between first-trimester serum vitamin A levels and the risk of developing GDM is of significant importance.

Regarding other pregnancy outcomes, the relationship between maternal vitamin A status and fetal growth remains ambiguous. While some studies correlate higher maternal or cord blood retinol with increased birth size or weight (39, 40), others associate high late-pregnancy levels with lower birth weight (41), and deficiencies in mid-to-late pregnancy have not been consistently linked to LBW (29). Maternal oxidative stress during pregnancy is known to contribute to low birth outcomes. As a key natural antioxidant, vitamin A can counteract oxygen free radicals and is hypothesized to exert a protective effect on fetal growth (42). For outcomes like emergency cesarean section, the potential link with maternal micronutrient status, including vitamin A, is plausible yet virtually unexplored. Hypothetically, suboptimal vitamin A levels might influence myometrial function, placental health, or fetal distress susceptibility—factors contributing to unplanned surgical delivery—but direct evidence is lacking. In summary, significant gaps persist in understanding the role of first-trimester vitamin A in pregnancy outcomes. First, its role during the early stages of pregnancy remains insufficiently understood. Second, its relationship with fetal growth indicators is unclear. Third, its potential connection to intrapartum outcomes like emergency cesarean section remains speculative and uninvestigated. Most existing evidence comes from mid-to-late pregnancy, limiting insights into early predictive or causal roles. Therefore, this prospective cohort study aims to investigate the association between maternal serum vitamin A levels in the first trimester and the risk of key adverse pregnancy outcomes—specifically GDM, LBW, and emergency cesarean section—among Chinese women, thereby addressing these critical knowledge gaps.

## Materials and methods

### Participants

This hospital-based prospective cohort study was conducted in the Department of Gynecology and Obstetrics at Shenzhen Nanshan People's Hospital, Shenzhen, China. Pregnant women were prospectively recruited during their first prenatal visit at the outpatient clinic between February 1, 2019, and December 31, 2020. The study investigated the association between maternal serum vitamin A levels in early pregnancy and adverse pregnancy outcomes. The prospective design was characterized by: (1) predefined inclusion and exclusion criteria prior to recruitment; (2) active collection of blood samples specifically for vitamin A measurement during the first trimester (6–13 weeks of gestation), not utilizing retrospectively stored samples; and (3) planned and systematic ascertainment of pregnancy outcomes at delivery. A total of 1,349 pregnant women were initially enrolled. The inclusion criteria were (1) women aged 18 or older; (2) having a singleton pregnancy; (3) at 6–13 weeks of gestation; and (4) who had registered and plan to deliver in the above-mentioned hospital. The exclusion criteria were (1) having a multiple pregnancy (*n* = 7); (2) death of the infant (*n* = 3); (3) GDM could not be diagnosed (*n* = 1); (4) having pre-pregnancy diabetes or gestational diabetes diagnosed before 24 weeks (*n* = 2); (5) having pre-pregnancy hypertension (*n* = 2); (6) having hepatitis or impaired liver function (*n* = 58); (7) having nephritis or impaired kidney function (*n* = 7); (8) lost to follow-up (*n* = 1); (9) missing vitamin A laboratory test data (*n* = 70); or (10) missing other baseline data (*n* = 121). After the exclusion of 272 potential participants due to ineligibility, 1,077 pregnant women were included as participants. Ethical approval for studying this cohort was obtained from the Ethics Committee of Shenzhen Nanshan People's Hospital (No. 2019072644), and all participants signed an informed consent form. The study was conducted in accordance with the Declaration of Helsinki developed by the World Medical Association.

### Data collection

All enrolled pregnant women received regular prenatal care at our hospital until delivery. Data regarding the occurrence of pregnancy complications, mode of delivery, and neonatal outcomes were comprehensively recorded and verified through the hospital's information system. The baseline data were collected through questionnaire surveys and from the hospital's medical record system. The collected data comprised the participants' age, weight, height, educational level, smoking and alcohol consumption status during pregnancy, disease history, pregnancy history, family history, and other information, and serum retinol concentrations and other laboratory data at 6–13 weeks of pregnancy. Pre-pregnancy body mass index (BMI) was calculated from participant-reported pre-pregnancy weight and height. BMI was calculated as weight (kg) divided by the square of height (meters). The gestational age (weeks) at delivery is determined by the time from the last menstrual period to the delivery. The sex and basic physical characteristics of newborns were obtained from records in the hospital information system. The weight and length of newborns at birth were measured using an infant length and weight measuring instrument, and their head circumference were measured using a tape measure. It was measured twice and the average value was taken as the final result.

### Definition of mother and infant adverse pregnancy outcomes

GDM, emergency cesarean section, premature rupture of membranes (PROM), postpartum eclampsia, fetal distress, premature birth, macrosomia, LBW (Low Birth Weight infants), LGA (Large for Gestational Age infants), and SGA (Small for Gestational Age infants) babies were identified as adverse maternal and fetal pregnancy outcomes. According to the diagnostic criteria established by the International Association of Diabetes and Pregnancy Research Groups, the participants underwent a 75-g oral glucose tolerance test (OGTT) between 24 and 28 weeks of gestation. If the participants' results met any of the following criteria or any combination thereof, they were diagnosed with GDM: a fasting plasma glucose ≥5.1 mmol/L, an OGTT 1-h plasma glucose concentration ≥10.0 mmol/L, and an OGTT 2-h plasma glucose concentration ≥8.5 mmol/L (43). An emergency cesarean section is defined as the urgent surgical delivery of a fetus to preserve the health of the pregnant woman or ensure fetal survival; premature rupture of membranes is characterized by the spontaneous rupture of amniotic membranes prior to the onset of labor; postpartum eclampsia, a severe complication of hypertensive disorders in pregnancy, typically manifests between 24 h and 10 days following delivery; fetal distress denotes a condition in which the fetus experiences physiological compromise due to hypoxia or acidosis during intrauterine life; premature birth is defined as delivery occurring between 28 and less than 37 completed weeks of gestation. SGA and LGA were defined as birth weights below the 10th percentile or above the 90th percentile of the mean weight of other infants of the same gestational age, respectively (44). Newborns with a birth weight >4,000 g were defined as macrosomia, and those with a birth weight < 2,500 g were defined as LBW (45).

### Biochemical analysis

Between 6 and 13 weeks of gestation, during the first routine prenatal examination at Nanshan District People's Hospital in Shenzhen, 2 ml of fasting venous blood was collected from pregnant women using both sodium fluoride-containing anticoagulant tubes and non-anticoagulant tubes. The collection was performed by trained and certified technicians. Immediately after collection, blood samples were centrifuged at 3,500 rpm for 5 min at 4 °C. The resulting supernatant was then carefully transferred and aliquoted into cryotubes to prevent repeated freeze-thaw cycles. Aliquots were stored at −80 °C in the biological sample bank for future analysis.

The measurement of serum retinol levels is a common method for assessing vitamin A status in the body. Serum samples were prepared as follows. Stored serum was thawed at 4 °C. A sample (100 μL) was treated with an internal standard solution (100 μL) and methanol (100 μL), and the resulting mixture was vortexed for 2 min. Subsequently, the mixture was treated with hexyl hydride (800 μL), vortexed for 5 min to extract retinol, and then centrifuged at 12,000 rpm for 10 min. Next, 650 μL of the supernatant was transferred to another vessel and then taken to dryness under a flow of nitrogen gas. The solid residue was re-dissolved in acetonitrile (100 μL), and the resulting solution was vortexed for 60 s and then centrifuged at 12,000 rpm for 10 min. An aliquot of the supernatant was analyzed by high-performance liquid chromatography–tandem mass spectrometry (HPLC-MS/MS) to determine its concentration of retinol.

The HPLC-MS/MS method was based on that used in a previous study (46). Retinol standard (≥98% pure by HPLC) was purchased from Macklin (Product No. V830149). The internal standard, [^2^H_4_]-Vitamin A (purity ≥98% by HPLC), was obtained from Shanghai Zhenzhun Biological Technology Co., Ltd. A stock solution (Solution A) of 698.202 μmol/L was prepared by accurately weighing 2.000 mg (precision: 0.001 mg) of the vitamin A standard, dissolving it in acetonitrile, and diluting to a final volume of 10 ml. An intermediate working solution of 6.982 μmol/L was then obtained by diluting 1.0 ml of Solution A to 100 ml with acetonitrile. By further diluting this intermediate working solution with acetonitrile, a series of standard working solutions were prepared with vitamin A concentrations of 5.946, 2.973, 1.487, 0.595, 0.297, 0.149, and 0.059 μmol/L. Mobile phases (formic acid, ammonium formate, acetonitrile, and methanol) were purchased from Merck. HPLC-MS/MS analysis was performed using an AB ExionLC system coupled with a Triple Quad™ 4500 tandem mass spectrometer (AB Sciex, Framingham, MA). The instruments were controlled using Analyst^®^ software. Chromatographic separation of sample components was performed on a Waters HPLC CORTECS C18 Column (2.1 mm × 50 mm, 2.7 μm). The mobile phases comprised 0.1% formic acid + 0.01 mol/L ammonium formate + water (A) and 0.05% formic acid + methanol (B). The injection volume was 10 μL, and the column temperature was 40 °C. Chromatographic separation was performed at a flow rate of 0.6 mL/min using the following gradient elution: 0–0.5 min with A:B = 30:70; 0.5–2.0 min with A:B = 30:70; 2.0–4.5 min with A:B = 0:100; and 4.6–5.6 min with A:B = 30:70. Mass spectrometry was performed using atmospheric pressure chemical ionization and in multi-reaction monitoring mode. The calibration curve was constructed using the internal standard method. The prepared series of standard working solutions were analyzed consecutively over three days. Vitamin A showed good linearity within the concentration range of 0.059 to 5.946 μmol/L, with correlation coefficients (R) of ≥0.999. The accuracy, expressed as recovery rates, ranged from 90.1 to 113.2% across three batches of validation. For every 30 test specimens, place one blank sample, one low-value quality control sample and one high-value quality control sample respectively, and run them once.

### Classification of serum vitamin A levels

According to the World Health Organization (WHO) related standards (47), the serum retinol concentrations are defined as follows: less than 0.7 μmol/L: Vitamin A Deficiency(VAD); 0.7 to 1.05 μmol/L: Marginal Vitamin A Deficiency (MVAD); more than 1.05 μmol/L: Normal Vitamin A Status (VAN).

### Statistical analysis

The participants were divided into five groups based on quintiles of serum retinol concentrations. Quintiles of retinol were created based on the cohort-specific distribution to ensure approximately equal numbers of participants in each group, a common approach in prospective cohort studies (48, 49). Continuous data are expressed as means and standard deviations (SDs) or medians and interquartile ranges (IQRs), depending on their distribution. Categorical variables are expressed as frequencies and percentages. Between-group differences were determined by conducting Kruskal–Wallis tests or one-way analyses of variance for continuous variables and conducting chi-square tests for categorical variables. Correlation analysis was performed to investigate the relationships between serum vitamin A, serum vitamin C, and serum vitamin E concentrations and fasting, 1-h PG, and 2-h PG concentrations. A multivariate logistic regression model was used to investigate the relationship between retinol concentrations and the risk of GDM. Model 1 was not adjusted. Model 2 was adjusted for age and preconception BMI, these factors represent strong risk factors for GDM (49–52). Model 3 was adjusted for the variables that model 2 was adjusted for plus for education background, parity, smoking status, alcohol consumption, family history of diabetes, and family history of hypertension. Odds ratios (ORs) and 95% confidence intervals (CIs) were determined. The participants were divided into subgroups based on age and BMI with cut-off points of 35 years and 24 kg/m^2^, respectively. The reference basis was the definition of advanced maternal age (AMA) (50) and the determination of adult body weight in China (WS/T 428-2013). The first quintile of serum retinol concentrations was used as the reference group for logistical regression analysis. Restricted cubic spline (RCS) regression models with three nodes were used to describe the potential nonlinear relationship between serum retinol concentrations and GDM risk. All analyses were performed using SPSS 24.0 software (SPSS Inc., Chicago, IL, USA), wherein a two-sided *p*-value < 0.05 was considered to indicate a statistically significant difference. Graphs were produced using R version 3.0.3 software (The R Foundation for Statistical Computing, Vienna, Austria).

## Results

### Baseline characteristics of the participants

Table 1 shows the differences in the demographic characteristics and anthropometric measurements of the participants grouped by serum retinol concentration quintiles. The participants comprised 1,077 singleton pregnant women aged 29.50 (± 4.06) years with a BMI of 20.69 ± 2.56 kg/m^2^ (mean ± SD). The median (IQR) concentrations of serum retinol were 0.66 (0.58–0.78) μmol/L, and all concentrations were within the safe range for humans. The Q1 to Q5 groups exhibited statistically significant differences in maternal age (*p* = 0.001), 2-h PG concentration (*p* = 0.005), birth weight (*p* = 0.016), and head circumference of newborns (*p* = 0.010). In terms of maternal and infant pregnancy outcomes, the Q1 to Q5 groups exhibited significant differences in the incidence of GDM (*p* = 0.014), emergency cesarean section (*p* = 0.027), and LGA (*p* = 0.035). In the Q5 group, the incidence of emergency cesarean section (15.6%) and LGA (13.7%) was the highest, and the incidence of GDM (15.1%) was the lowest.

**Table 1:** Characteristics of participants according to quintiles of vitamin A (*N* = 1077).

characteristics		Vitamin A levels (μmol/L)					*P* value
	Total	Quintile 1	Quintile 2	Quintile 3	Quintile 4	Quintile 5	
		≤0.57	0.57–0.63	0.63–0.69	0.69–0.82	0.82–2.50	
No. of maternal cases	1,077	243	206	207	209	212	
Maternal age (years)	29.50 ± 4.06	30.45 ± 4.46	29.85 ± 4.02	28.72 ± 3.56	28.94 ± 4.07	29.40 ± 3.84	0.001
Age categories [*n* (%)]							
< 35 [*n* (%)]	939 (87.2)	190 (78.2)	179 (86.9)	194 (93.7)	187 (89.5)	189 (89.2)	0.000
≥35 [*n* (%)]	138 (12.8)	53 (21.8)	27 (13.1)	13 (6.3)	22 (10.5)	23 (10.8)	
BMI (kg/m^2^)	20.69 ± 2.56	20.66 ± 2.38	20.88 ± 2.65	20.40 ± 2.37	20.72 ± 2.70	20.79 ± 2.68	0.391
BMI categories [*n* (%)]							
< 24 [*n* (%)]	955 (88.7)	216 (88.9)	177 (85.9)	188 (90.8)	186 (89.0)	188 (88.7)	0.638
≥24 [*n* (%)]	122 (11.3)	27 (11.1)	29 (14.1)	19 (9.2)	23 (11.0)	24 (11.3)	
Education background [*n* (%)]							
Primary [*n* (%)]	84 (7.8)	17 (7.0)	16 (7.8)	14 (6.8)	22 (10.5)	15 (7.1)	0.647
Secondary [*n* (%)]	166 (15.4)	32 (13.2)	28 (13.6)	35 (16.9)	37 (17.7)	34 (16.0)	
College or above [*n* (%)]	827 (76.8)	194 (79.8)	162 (78.6)	158 (76.3)	150 (71.8)	163 (76.9)	
Parity [*n* (%)]							
Primiparity [*n* (%)]	649 (60.3)	139 (57.2)	122 (59.2)	139 (67.1)	127 (60.8)	122 (57.5)	0.213
Multiparity [*n* (%)]	428 (39.7)	104 (42.8)	84 (40.8)	68 (32.9)	82 (39.2)	90 (42.5)	
History of miscarriage [*n* (%)]	278 (25.8)	66 (27.2)	56 (27.2)	45 (21.7)	51 (24.4)	60 (28.3)	0.536
retinol (μmol/L)	0.66 (0.58–0.78)	0.55 (0.52–0.56)	0.60 (0.59–0.61)	0.67 (0.65–0.68)	0.74 (0.71–0.79)	0.93 (0.87–1.09)	0.001
Initial inspection blood pressure							
SBP (mm Hg)	110.42 ± 10.56	110.10 ± 10.27	111.19 ± 10.99	109.47 ± 10.52	111.29 ± 10.66	109.19 ± 10.99	0.410
DBP (mm Hg)	66.64 ± 7.94	66.79 ± 7.91	66.49 ± 8.04	65.63 ± 7.89	67.12 ± 8.03	66.50 ± 7.98	0.596
OGTT glucose metabolism (mmol/L)							
Fasting plasma glucose (mmol/L)	4.54 ± 0.33	4.56 ± 0.34	4.55 ± 0.35	4.53 ± 0.30	4.57 ± 0.34	4.52 ± 0.31	0.455
1-h post-load glucose (mmol/L)	7.98 ± 1.62	8.06 ± 1.62	8.09 ± 1.79	7.99 ± 1.63	7.99 ± 1.61	7.81 ± 1.46	0.562
2-h post-load glucose (mmol/L)	6.98 ± 1.39	7.15 ± 1.40	7.16 ± 1.43	6.76 ± 1.36	7.06 ± 1.44	6.78 ± 1.30	0.005
Birth weight of newborn (g)	3,274.44 ± 452.62	3,209.32 ± 439.63	3,286.63 ± 484.11	3,248.42 ± 421.69	3,265.35 ± 445.81	3,370.91 ± 459.34	0.016
Birth length of newborn (cm)	49.91 ± 1.85	49.72 ± 1.76	49.93 ± 2.00	49.86 ± 1.65	49.88 ± 2.11	50.18 ± 1.71	0.215
Birth head circumference of newborn (cm)	34.23 ± 1.49	34.12 ± 1.43	34.25 ± 1.72	34.09 ± 1.28	34.10 ± 1.65	34.63 ± 1.29	0.010
Adverse maternal and fetal outcomes							
GDM [*n* (%)]	241 (22.4)	67 (27.6)	47 (22.8)	41 (19.8)	54 (25.8)	32 (15.1)	0.014
Emergency cesarean section [*n* (%)]	108 (10.0)	19 (7.8)	15 (7.3)	23 (11.1)	18 (8.6)	33 (15.6)	0.027
PROM [*n* (%)]	176 (16.3)	40 (16.5)	32 (15.5)	30 (14.5)	41 (19.6)	33 (15.6)	0.671
Postpartum eclampsia [*n* (%)]	14 (1.3)	5 (2.1)	4 (1.9)	2 (1.0)	2 (1.0)	1 (0.5)	0.531
Fetal intrauterine distress [*n* (%)]	60 (5.6)	20 (8.2)	12 (5.8)	9 (4.3)	11 (5.3)	8 (3.8)	0.265
Premature birth [*n* (%)]	46 (4.3)	12 (4.9)	10 (4.9)	8 (3.9)	10 (4.8)	6 (2.8)	0.785
Macrosomia [*n* (%)]	31 (2.9)	7 (2.9)	5 (2.4)	5 (2.4)	3 (1.4)	11 (5.2)	0.206
LBW [*n* (%)]	37 (3.4)	13 (5.3)	6 (2.9)	6 (2.9)	9 (4.3)	3 (1.4)	0.190
SGA [*n* (%)]	65 (6.0)	20 (8.2)	15 (7.3)	12 (5.8)	6 (2.9)	12 (5.7)	0.172
LGA [*n* (%)]	105 (9.7)	20 (8.2)	26 (12.6)	18 (8.7)	12 (5.7)	29 (13.7)	0.035

BMI: body mass index; SBP, systolic blood pressure; DBP, diastolic blood pressure; OGTT, oral glucose tolerance test; GDM, gestational diabetes mellitus; PROM, premature rupture of membranes; LBW, low-birth-weight infants; PTB, preterm birth; SGA, small-for-gestational-age infants; LGA, large-for-gestational-age infants.

### Associations between serum vitamin A levels during the first trimester and risk of GDM

A negative correlation was observed between serum retinol concentrations and 1-h and 2-h PG concentrations (Table S2). Furthermore, the logistic regression model revealed the relationship between serum retinol concentrations in early pregnancy and the risk of GDM (Table 2). In model 1, the participants with serum retinol concentrations in Q5 (0.82 < Q5 ≤ 2.50 μmol/L) had lower odds of GDM than those with serum retinol concentrations in Q1 ( ≤ 0.57 μmol/L) (OR: 0.47; 95% CI: 0.29–0.75). In models 2 and 3, after adjusting for confounding factors, the correlation between serum retinol concentrations and GDM remained significant (OR: 0.51; 95% CI: 0.31–0.82 and OR: 0.50; 95% CI: 0.31–0.81, respectively). RCS analysis revealed no significant nonlinear relationship between serum retinol levels and the risk of GDM (Supplementary Figure S1A) (*P* for nonlinearity > 0.05).

**Table 2:** Odds ratios (95% confidence intervals) for the occurrence of GDM according to the vitamin A levels at the first trimester^*^.

Variables	Vitamin A levels (μmol/L)					*P trend*
	Quintile 1	Quintile 2	Quintile 3	Quintile 4	Quintile 5	
	≤0.57	0.57–0.63	0.63–0.69	0.69–0.82	0.82–2.50	
GDM						
Case/*N*	67/243	47/206	41/207	54/209	32/212	
Model 1	1.00	0.78 (0.51, 1.19)	0.65 (0.42, 1.01)	0.91 (0.60, 1.39)	0.47^**^ (0.29, 0.75)	0.006
Model 2	1.00	0.82 (0.53, 1.28)	0.79 (0.50, 1.24)	1.03 (0.67, 1.59)	0.51^**^ (0.31, 0.82)	0.018
Model 3	1.00	0.81 (0.52, 1.27)	0.78 (0.49, 1.25)	1.02 (0.66, 1.59)	0.50^**^ (0.31, 0.81)	0.015

^*^p < 0.05, ^**^ *p* < 0.01. GDM, gestational diabetes mellitus. Model 1: without adjustment. Model 2: adjusted for age, BMI; Model 3 :adjusted for the variables in Model 1: plus education background, smoking status, alcohol status, parity, history of miscarriage, nation, history of diabetes, history of hypertension.

**Figure 1:**
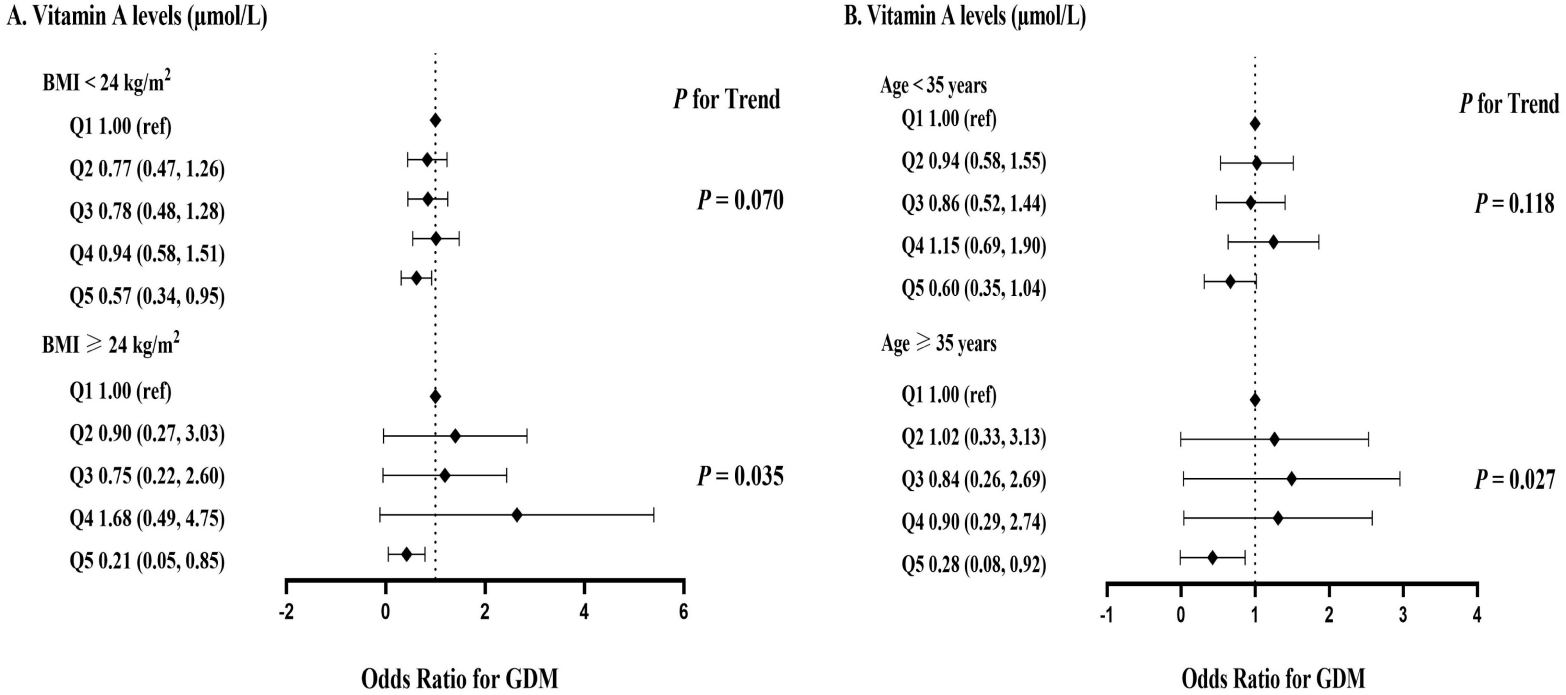
ORs (95% CIs) for serum vitamin A levels associated with GDM according to different BMI ang age values. The model adjusted education background, smoking status, alcohol status, parity, gravidity, history of miscarriage, nation, history of diabetes, history of hypertension.

The ORs (95% CIs) associated with serum retinol concentrations and GDM in participants classified by BMI or age are shown in Figure 1. In the BMI ≥24 kg/m^2^ group, high serum retinol concentrations were associated with low odds of GDM (*p*-trend = 0.035); specifically, participants in the highest quintile (Q5) had significantly lower odds compared to the lowest quintile (adjusted OR: 0.21, 95% CI: 0.05–0.85). This trend was not observed in the BMI < 24 kg/m^2^ group. In the aged ≥35 group, high serum retinol concentrations were associated with low odds of GDM (*p*-trend = 0.027; Q5 adjusted OR: 0.28, 95% CI: 0.08–0.92), whereas no significant trend was observed in the aged < 35 years group (*p*-trend = 0.118; Q5 adjusted OR: 0.60, 95% CI: 0.35–1.04).

### Association between serum vitamin A levels in early pregnancy and risk of other adverse pregnancy outcomes

Table 3 shows the relationship between serum retinol concentrations in early pregnancy and some adverse pregnancy outcomes. After adjustment for age and BMI, the risk of fetal distress in utero decreased in the Q5 group as serum retinol concentrations increased (OR: 0.41; 95% CI: 0.18–0.96). Compared with the Q1 group, the Q5 group had increased odds of emergency cesarean section in model 1 (OR: 2.17; 95% CI: 1.20–3.95, *p*-trend = 0.003). The same trend existed after adjustment for potential confounding factors, i.e., in model 2 (OR: 2.26; 95% CI: 1.24–4.13, *p*-trend = 0.003) and model 3 (OR: 2.31; 95% CI: 1.26–4.26, *p*-trend = 0.002). In Model 3 (the fully adjusted model), higher serum retinol concentrations were associated with a reduced odds ratio for low birth weight (LBW): the odds of LBW in the highest quintile (Q5) were lower than those in the lowest quintile (Q1) (OR: 0.26; 95% CI: 0.07–0.96), although the increasing trend in odds ratios from Q1 to Q5 was not statistically significant (*p* for trend = 0.099). In model 2, group Q5 had higher odds of LGA than group Q1 (OR: 1.92; 95% CI: 1.04–3.54). The odds of SGA in the Q4 group were significantly lower than that in the Q1 group (OR: 0.33; 95% CI: 0.13–0.84), and this trend persisted after adjustment for potential confounding factors, i.e., in model 3 (OR: 0.32; 95% CI: 0.12–0.82). However, there was no significant association between serum retinol concentrations and the odds of other adverse pregnancy outcomes (Supplementary Table S3, Supplementary Figure S2). RCS analysis indicated no significant nonlinear relationships between serum retinol levels and the risks of emergency cesarean section (Supplementary Figure S1B), intrauterine fetal distress (Supplementary Figure S1C), or small for gestational age (Supplementary Figure S1D) (*P* for nonlinearity > 0.05).

**Table 3:** ORs (95% CIs) for adverse pregnancy outcomes according to the vitamin A levels at the first trimester.

Variables	Vitamin A levels (μmol/L)					*P* trend
	Quintile 1	Quintile 2	Quintile 3	Quintile 4	Quintile 5	
	≤ 0.57	0.57–0.63	0.63–0.69	0.69–0.82	0.82–2.50	
Fetal distress in utero						
Case/*N*	20/243	12/206	9/207	11/209	8/212	
Model 1	1.00	0.69 (0.33–1.45)	0.51 (0.23–1.14)	0.62 (0.29–1.33)	0.44 (0.19, 1.01)	0.068
Model 2	1.00	0.67 (0.32, 1.40)	0.47 (0.21, 1.05)	0.58 (0.27, 1.26)	0.41^*^ (0.18, 0.96)	0.054
Model 3	1.00	0.71 (0.33, 1.51)	0.47 (0.21, 1.08)	0.60 (0.28, 1.31)	0.44 (0.19, 1.04)	0.073
Emergency cesarean section						
Case/*N*	19/243	15/206	23/207	18/209	33/212	
Model 1	1.00	0.93 (0.46, 1.87)	1.47 (0.78, 2.79)	1.11 (0.57, 2.18)	2.17^*^ (1.20, 3.95)	0.003
Model 2	1.00	0.94 (0.46, 1.91)	1.58 (0.82, 3.01)	1.15 (0.59, 2.27)	2.26^**^ (1.24, 4.13)	0.003
Model 3	1.00	0.95 (0.46, 1.93)	1.53 (0.79, 2.96)	1.18 (0.60, 2.34)	2.31^**^ (1.26, 4.26)	0.002
LBW						
Case/*N*	13/243	6/206	6/207	9/209	3/212	
Model 1	1.00	0.53 (0.20, 1.42)	0.53 (0.20, 1.42)	0.80 (0.33, 1.90)	0.25^*^ (0.07, 0.90)	0.070
Model 2	1.00	0.57 (0.21, 1.53)	0.57 (0.21, 1.55)	0.84 (0.35, 2.03)	0.27^*^ (0.08, 0.97)	0.088
Model 3	1.00	0.52 (0.19, 1.45)	0.59 (0.21, 1.65)	0.88 (0.35, 2.17)	0.26^*^ (0.07, 0.96)	0.099
SGA						
Case/*N*	20/243	15/206	12/207	6/209	12/212	
Model 1	1.00	0.88 (0.44, 1.76)	0.69 (0.33, 1.44)	0.33^*^ (0.13, 0.84)	0.67 (0.32, 1.40)	0.156
Model 2	1.00	0.88 (0.44, 1.79)	0.64 (0.30, 1.36)	0.32^*^ (0.13, 0.82)	0.65 (0.31, 1.37)	0.137
Model 3	1.00	0.90 (0.44, 1.84)	0.64 (0.30, 1.37)	0.32^*^ (0.12, 0.82)	0.70 (0.33, 1.48)	0.185
LGA						
Case/*N*	20/243	26/206	18/207	12/209	29/212	
Model 1	1.00	1.61 (0.87, 2.98)	1.06 (0.55, 2.07)	0.68 (0.32, 1.43)	1.77 (0.97, 3.23)	0.217
Model 2	1.00	1.66 (0.89, 3.11)	1.22 (0.62, 2.42)	0.72 (0.34, 1.53)	1.92^*^ (1.04, 3.54)	0.152
Model 3	1.00	1.65 (0.88, 3.10)	1.29 (0.65, 2.57)	0.72 (0.34, 1.53)	1.79 (0.96, 3.33)	0.256

^*^p < 0.05, ^**^ *p* < 0.01. Model 1: without adjustment. Model 2: adjusted for age, BMI; Model 3: adjusted for the variables in Model 1 plus education background, smoking status, alcohol status, parity, history of miscarriage, nation, history of diabetes, history of hypertension. LBW, low-birth-weight infants; SGA, small-for-gestational-age infants; LGA, large-for-gestational-age infants.

## Discussion

In this prospective cohort study, the associations between maternal serum vitamin A levels in early pregnancy and adverse pregnancy outcomes were determined. None of the participants had excessive serum retinol concentrations. Increased serum retinol concentrations were found to be significantly associated with decreased risks of GDM, LBW, and SGA and an increased risk of emergency cesarean delivery. Consistent with the aforementioned findings, serum retinol concentration as a continuous variable was negatively correlated with 1-h and 2-h PG concentrations. In model 2, serum retinol concentrations in the Q5 group were significantly associated with a decreased risk of intrauterine fetal distress and a significantly increased risk of LGA. No significant association was observed between serum retinol concentrations and other adverse pregnancy outcomes.

Vitamin A is a fat-soluble vitamin and is an essential micronutrient for the human body, vitamin A also plays a key role in the development of pregnant women and fetuses (11). Moreover, vitamin A has antioxidant properties, and previous research has shown that oxidative stress may lead to the development of GDM (24). Therefore, we evaluated the association of maternal serum vitamin A levels in early pregnancy with the occurrence of GDM and other adverse pregnancy outcomes. Only a few studies have assessed the relationship between vitamin A (in terms of food intake or physical conditions) and the risk of GDM, and they have reported inconsistent results. Our findings are similar to those of a recent case–control study in China (Supplementary Table S1), which found that serum vitamin A levels were lower in the group with GDM than in the group without GDM (51). However, it is difficult to determine a causal relationship between serum vitamin A levels and GDM based on the aforementioned results because the testing of serum retinol concentrations and diagnosis of GDM were performed in pregnant women at 24–28 weeks. A case–control study conducted in Iran of pregnant women at 30–32 weeks showed that retinol concentrations in the GDM group were significantly lower than those in the control group (31). It does not demonstrate a causal relationship between gestational diabetes mellitus (GDM) and decreased retinol levels, since the fat-soluble vitamins were measured after the confirmation of GDM in the women. However, a previous retrospective cohort study in China showed that serum vitamin A levels in the GDM group were higher than those in the non-GDM group in the first trimester (52). In addition, a multiple logistic regression analysis in this study did not find that vitamin A was an independent influencing factor for GDM, which is inconsistent with the findings in the present study (52). Similarly, another recent Chinese prospective cohort study found that increases in serum vitamin A levels in early pregnancy led to increases in the risk of GDM, which is inconsistent with the findings in the present study (38). The discrepancies between these studies and ours may be attributed to differences in study populations, timing of biomarker assessment, and, importantly, variations in the confounding factors considered during adjustment, particularly those reflecting overall nutritional and inflammatory status. However, unlike the present study, despite the fact that vitamin A is stored and metabolized in the liver, the aforementioned two studies did not exclude pregnant women with hepatitis or impaired liver function. Furthermore, it was previously found that women with GDM may experience mild liver dysfunction (53), potentially leading to higher serum concentrations of retinol in the GDM group than in the non-GDM group. There are studies that show that common infections can increase the risk of vitamin A deficiency by decreasing intake, decreasing absorption, and increasing excretion (54). Consequently, serum retinol concentration is a composite biomarker influenced by both nutritional intake and systemic inflammation. The associations observed in our study between vitamin A and pregnancy outcomes, including GDM, must be interpreted within this physiological context, where retinol levels may serve as a proxy for a broader favorable nutritional and anti-inflammatory status rather than indicating a direct causal role for vitamin A itself. Studies have shown that retinol exhibits a significant inverse correlation with pro-inflammatory compounds such as IL-8 in both maternal and umbilical cord blood, highlighting its role as a biomarker embedded within inflammatory networks. Conversely, the positive correlation between placental retinol and anti-inflammatory cytokines such as IL-10 further underscores its link to immune balance rather than an isolated causal pathway (55).

The present study found that participants aged 35 or older or with a BMI of 24 kg/m^2^ or higher had higher serum retinol concentrations and a lower risk of GDM than other participants. Women of advanced maternal age are those who will be aged 35 or older at their estimated date of delivery (50). Some studies have demonstrated that AMA is associated with the occurrence of GDM (56, 57). In addition, the risk of GDM is higher in women with obesity than in those with a normal BMI (58, 59). Notably, in the present study, this theoretically higher-risk population exhibited higher serum retinol levels along with a lower risk of GDM. This observation suggests that adequate vitamin A status, potentially reflecting a more favorable overall nutritional and metabolic profile, may play a modulating role in mitigating the age- and obesity-related risk pathways for GDM. As an antioxidant, vitamin A can alleviate oxidative stress, which is a key contributor to the pathophysiology of GDM and is often exacerbated in older and obese pregnant individuals (19, 20, 22, 23, 60, 61). However, this association similarly needs to be interpreted in the context of overall nutrition and inflammation, as the higher retinol levels in these groups might reflect a more robust nutritional reserve or a less pronounced inflammatory state, which collectively contribute to metabolic health. Therefore, it is important to monitor serum vitamin A concentrations in these two populations of women during the first trimester to reduce the incidence of GDM.

A deficiency of vitamin A is harmful to the mother and fetus, while an excess of vitamin A can have toxic and teratogenic effects early in pregnancy (62, 63). A prospective cohort study conducted in Chengdu, China, showed that dietary intake of vitamin A during the first trimester was associated with the development of GDM, it also showed that a higher dietary intake of vitamin A than the recommended nutrient intake reduced the risk of GDM (64). Another case–control study in a Chinese population examined dietary nutrition patterns and assessed their association with GDM risk, it found that a vitamin nutrition pattern diet (a diet rich in vitamin A, carotene, vitamin B2, vitamin B6, vitamin C, dietary fiber, folate, calcium, and potassium) was associated with a reduced risk of GDM (65). This pattern strongly supports the view that the benefits attributed to vitamin A in observational studies may arise from its integrated role within a broader context of favorable nutrients and dietary patterns. Therefore, the observed association with serum vitamin A likely reflects the advantage conferred by its participation as a component of a comprehensively beneficial nutritional profile and dietary practice. In practice, pregnant women should be directed to eat a moderate amount of foods rich in vitamin A, because vitamin A may improve the pregnancy outcomes of mothers and babies. The present study did not perform surveys of dietary intake of vitamin A, in the future, dietary investigations can be supplemented to distinguish the dietary sources of vitamin A and further refine the analysis of the association between vitamin A and adverse pregnancy outcomes.

Some studies have found that women with GDM have an increased risk of adverse perinatal outcomes, such as macrosomia and LGA, and that GDM is a risk factor for emergency cesarean section (66, 67). Although cesarean delivery has progressively become one of the criteria for disease evaluation, it should not be adopted as a study endpoint for gestational diabetes mellitus (GDM) since the procedure is associated not only with disease morbidity but also influenced by physician decision-making and the standard of clinical practice. Dittakarn et al. (68) found that the incidence of emergency cesarean delivery was significantly greater in pregnant women with GDM than in normal pregnant women. In the present study, high serum vitamin A levels were associated with a decreased risk of GDM but with an increased risk of emergency cesarean section and LGA. The association between serum vitamin A levels and emergency cesarean section remains poorly understood. This paradoxical association further exemplifies the complexity of interpreting serum retinol levels; a marker of favorable metabolic status for one outcome (GDM) may be linked to other, multifactorial clinical decisions or pathways (cesarean section) in ways that are not directly causal. Mechanistically, while our study participants did not have excessive retinol levels, the observed association might be partly explained by vitamin A's dose-sensitive role in processes critical for parturition. High doses of vitamin A are recognized teratogens, with case reports linking excessive intake during early pregnancy to congenital anomalies, underscoring its potent influence on fetal development (69). This teratogenic potential implies that vitamin A can critically modulate placental development and function. Even at non-teratogenic levels, variations in vitamin A status may still influence placental vascular development and function, potentially affecting fetal wellbeing and labor progression. This study did not account for potential confounding factors, such as fetal position, that may influence the occurrence of emergency cesarean section; therefore, further investigation is warranted in future research.

Maternal vitamin A status plays a crucial role in the normal development of the fetus and neonate (70). A low intake of vitamin A has been linked to LBW and SGA (71, 72). In the present study, although there was no significant difference in the incidence of LBW across the five quintiles based on serum retinol concentrations, a logistic regression analysis revealed that high vitamin A levels were associated with a decreased risk of LBW. Therefore, this association may reflect the role of vitamin A as part of an overall favorable nutritional environment rather than its independent effect. This interpretation aligns with findings that deficiencies of vitamin A and other micronutrients frequently coexist and are collectively linked to adverse outcomes like anemia, underscoring the interconnected nature of nutritional status (73). A previous study conducted in China found that infant umbilical cord blood retinol concentrations were significantly and positively correlated with birth weight (74). However, this study did not measure serum retinol concentrations in pregnant mothers. Additionally, a cluster-randomized placebo-controlled trial conducted in rural northwestern Bangladesh found that prenatal vitamin A or β-carotene supplementation did not result in a decrease in the incidence of LBW (75). The heterogeneous nature of results from previous studies may be attributable to variations in study design and populations, as well as to differences in the overall nutritional context and confounding factors considered. Furthermore, a retrospective cohort study by Catov et al. suggested that regular periconceptional multivitamin use was associated with a reduced risk of non-overweight women having SGA newborns (76), but did not analyse the effects of serum concentrations of retinol on SGA. The findings of the present study indicate that high serum concentrations of retinol (0.69 μmol/L −0.82 μmol/L) were associated with a decreased incidence of SGA (OR: 0.32, 95% CI: 0.12–0.82). We interpret this finding with caution, positing that adequate vitamin A status may merely serve as a marker or exert a synergistic effect with other nutritional factors essential for fetal growth, such as iron and folate. This study found that higher vitamin A levels were associated with both protective effects (reduced risk of GDM and LBW) and adverse effects (increase emergency cesarean section rates). While this appears paradoxical, it may be attributable to the complexity of vitamin A's biological functions, distinct pathophysiological pathways for different outcomes, and threshold effects specific to certain gestational periods. Crucially, this dual nature reinforces the overarching concept that retinol serves as a versatile biomarker that is responsive to and reflective of diverse, sometimes opposing, physiological states, rather than a unidirectional causal agent. Although this study observed associations between vitamin A levels and certain outcomes, the underlying mechanisms are likely to be indirect, involving the support of placental development and participation in immune regulation. Mechanistic studies support this: animal models of vitamin A deficiency demonstrate impaired placental growth, abnormal histology, and increased inflammatory markers such as neutrophil infiltration and elevated TNFα expression (77). Critically, specific nuclear receptors for vitamin A (e.g., RXR) are essential for normal placental labyrinthine zone development (78). Concurrently, vitamin A is a known immunomodulator that can alter the balance between Th1/Th2 responses and suppress pro-inflammatory responses (79). The complexity of its role is further corroborated by human studies indicating that, at the placental site, retinol levels are inversely associated with pro-inflammatory cytokines such as IL-8 and positively associated with anti-inflammatory cytokines such as IL-10. This suggests that the serum retinol levels observed in mothers may not directly drive pregnancy outcomes but instead reflect the state of a broader, balanced inflammatory microenvironment at the maternal-fetal interface, which is crucial for maintaining a healthy pregnancy. Furthermore, the placenta may serve as a key site that integrates nutrient levels and inflammatory signals, potentially acting as a protective buffer for the fetus (55).

This study has several limitations. First, the lack of systematically collected data on detailed dietary vitamin A intake and supplement use, coupled with the single measurement of vitamin A levels only in early pregnancy, limits our ability to distinguish the sources of exposure and assess the dynamic changes in vitamin A status throughout gestation. Second, although strong risk factors such as preconception BMI were adjusted for, gestational weight gain (GWG) was not included as a covariate in the primary models. It is noteworthy that GWG, particularly occurring in the mid-to-late stages of pregnancy, follows the key exposure variable (first-trimester vitamin A level) in time; therefore, treating it as a confounder requires careful consideration of temporal causality. In addition, we lacked data on physical activity during pregnancy, other critical micronutrients (such as folate, vitamin B12, vitamin D, and iron), and inflammatory markers, which precludes ruling out residual confounding. Serum retinol is a composite biomarker reflecting overall nutritional and inflammatory status; thus, the observed associations may arise from a broader nutritional milieu rather than a specific, independent causal effect of vitamin A. Third, prepregnancy height and weight were self-reported, which may introduce measurement bias. Fourth, as a single-center study, the generalizability of our findings to broader populations requires further verification. Fifth, only serum retinol concentrations were measured, while related markers such as oxidative stress were not assessed concurrently. Finally, the single time-point measurement in early pregnancy may not adequately capture the dynamic changes in vitamin A status influenced by physiological alterations such as blood volume expansion, increased fetal demands, and dietary shifts during mid- and late gestation, potentially underestimating its cumulative effect. Future research should incorporate longitudinal designs, multi-nutrient models, and more comprehensive assessment of exposures and confounding factors to further elucidate the role of vitamin A in pregnancy health.

## Conclusions

In this prospective cohort study of Chinese women, first-trimester maternal serum retinol levels were associated with the risks of key adverse pregnancy outcomes. Higher levels were associated with a reduced risk of gestational diabetes mellitus and low birth weight, but with an increased risk of emergency cesarean delivery. These findings highlight the complex relationship between vitamin A status and pregnancy outcomes.

## Data Availability

The raw data supporting the conclusions of this article will be made available by the authors, without undue reservation.
