# Supplementary material for: Association of maternal serum vitamin a levels in the first trimester with the risk of adverse pregnancy outcomes: a prospective cohort study of Chinese women
Source: Front Nutr. 2026 Apr 2;13:1735875. doi: 10.3389/fnut.2026.1735875 (PMC13082939; doi:10.3389/fnut.2026.1735875)
Supplement: Supplementary file 1 [file Supplementary_file_1.zip › Yuan_VitA_AdversePregnancy_Supplementary Figures and Tables/Supplementary Figure 2.docx]

Supplementary Material


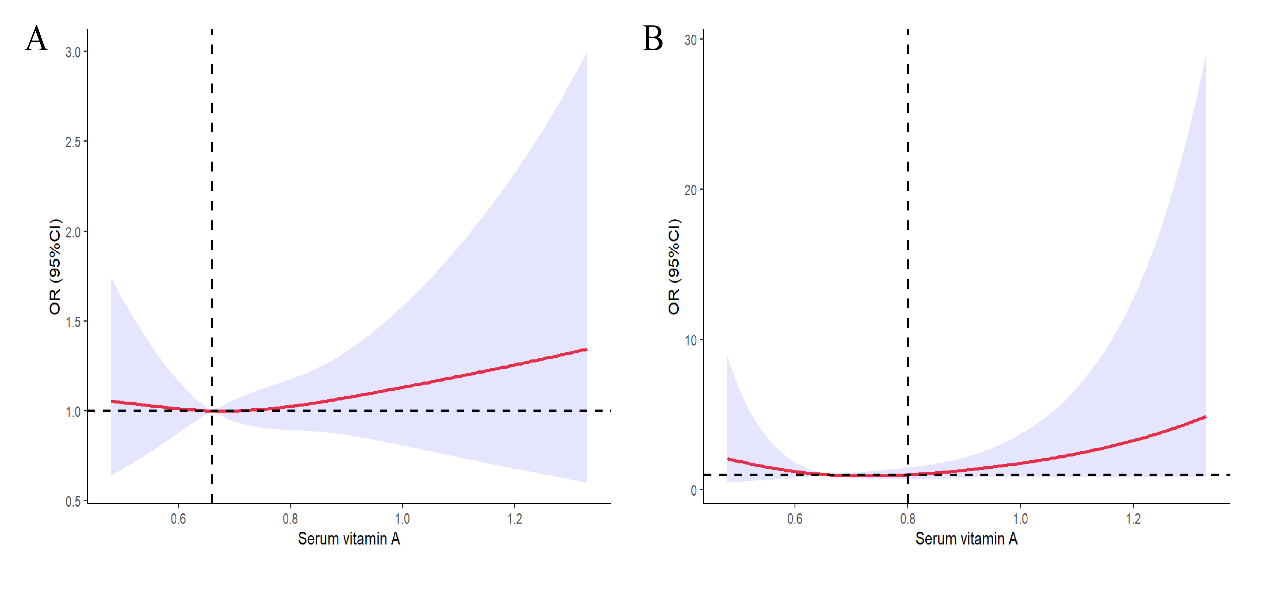

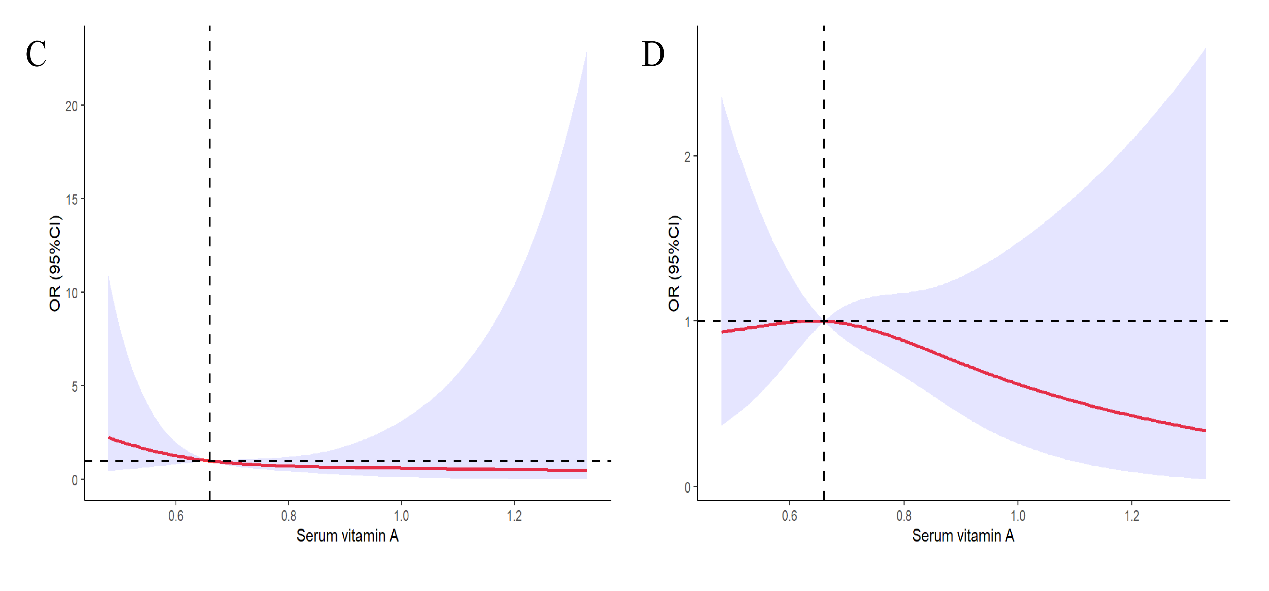

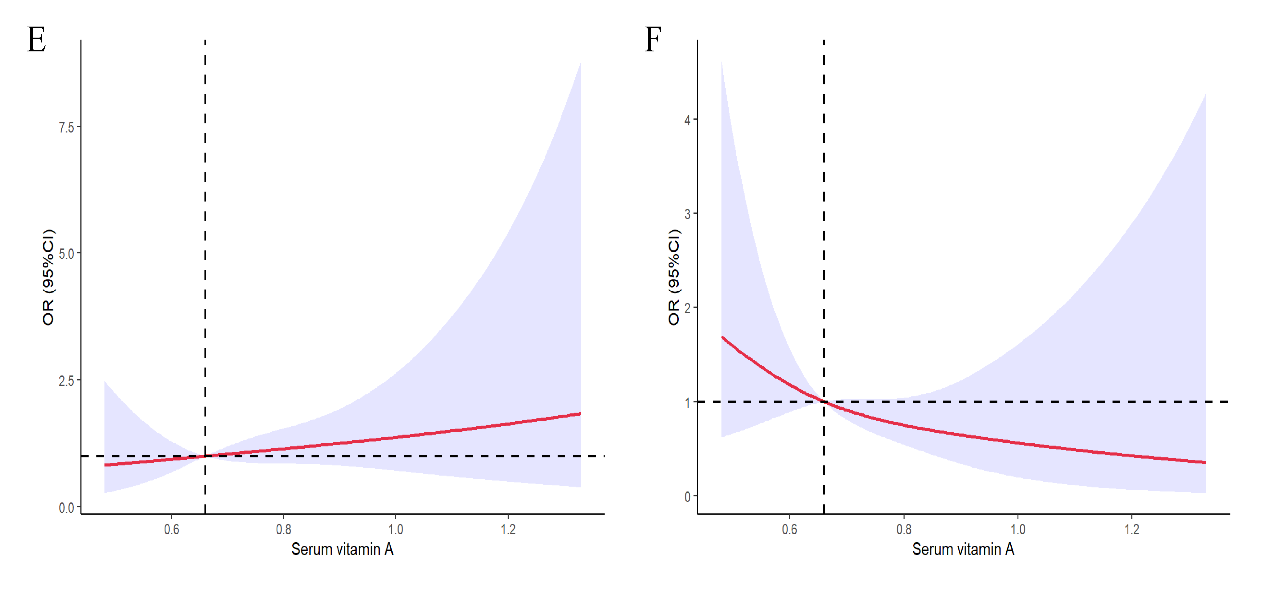

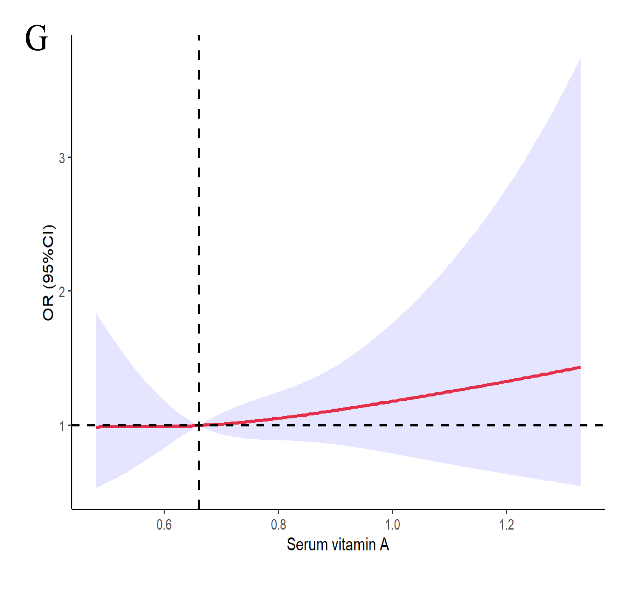


**Supplementary Figure 1.** RCS regression analysis of serum vitamin A levels with other adverse maternal and foetal outcomes. (A) PROM: premature rupture of membranes, (B) postpartum hemorrhage, (C) postpartum eclampsia, (D) premature birth，(E) macrosomia, (F) LBW: low-birth-weight infants; (G) LGA: large for gestational age infants. The median of vitamin A 0.66 µmol/L and 0.8 µmol/L was selected as the reference levels. The lines indicate estimated ORs, and the light blue-shaded areas represent 95%CI.
