# Supplementary material for: Association of maternal serum vitamin a levels in the first trimester with the risk of adverse pregnancy outcomes: a prospective cohort study of Chinese women
Source: Front Nutr. 2026 Apr 2;13:1735875. doi: 10.3389/fnut.2026.1735875 (PMC13082939; doi:10.3389/fnut.2026.1735875)
Supplement: Supplementary file 1 [file Supplementary_file_1.zip › Yuan_VitA_AdversePregnancy_Supplementary Figures and Tables/Supplementary Table 1.docx]

Supplementary Material

**Supplementary Table 1.** Serum vitamin A concentrations of women with GDM and non-GDM (N= 1077).

| Characteristics | GDM (n ₌ 241) | Non-GDM (n ₌ 836) | P value |
| --- | --- | --- | --- |
| vitamin A (µmol/L) | 0.65 (0.57 - 0.74) | 0.67 (0.59 - 0.80) | 0.039 |
