# Supplementary material for: Association of maternal serum vitamin a levels in the first trimester with the risk of adverse pregnancy outcomes: a prospective cohort study of Chinese women
Source: Front Nutr. 2026 Apr 2;13:1735875. doi: 10.3389/fnut.2026.1735875 (PMC13082939; doi:10.3389/fnut.2026.1735875)
Supplement: Supplementary file 1 [file Supplementary_file_1.zip › Yuan_VitA_AdversePregnancy_Supplementary Figures and Tables/Supplementary Table 3.docx]

Supplementary Material

**Supplementary Table 3.** ORs (95% CIs) for adverse pregnancy outcomes according to the Vitamin A levels at the first trimester.

| Variables | Vitamin A levels (µmol/L) | | | | |  |
| --- | --- | --- | --- | --- | --- | --- |
|  | Quintile 1 | Quintile 2 | Quintile 3 | Quintile 4 | Quintile 5 | *P* trend |
|  | ≤ 0.57 | 0.57 - 0.63 | 0.63 - 0.69 | 0.69 - 0.82 | 0.82 - 2.50 |  |
| PROM |  |  |  |  |  |  |
| Case/N | 40/243 | 32/206 | 30/207 | 41/209 | 33/212 |  |
| Model 1 | 1.00 | 0.93 (0.56, 1.55) | 0.86 (0.51, 1.44) | 1.24 (0.77,2.00) | 0.94 (0.57, 1.55) | 0.934 |
| Model 2 | 1.00 | 0.97 (0.58, 1.61) | 0.95 (0.56, 1.60) | 1.31 (0.80, 2.13) | 0.99 (0.60, 1.65) | 0.786 |
| Model 3 | 1.00 | 0.95 (0.57, 1.59) | 0.94 (0.55, 1.59) | 1.33 (0.81, 2.17) | 1.00 (0.60, 1.67) | 0.728 |
| Postpartum eclampsia |  |  |  |  |  |  |
| Case/N | 5/243 | 4/206 | 2/207 | 2/209 | 1/212 |  |
| Model 1 | 1.00 | 0.94 (0.25, 3.56) | 0.46 (0.09, 2.42) | 0.46 (0.09, 2.40) | 0.23 (0.03, 1.95) | 0.110 |
| Model 2 | 1.00 | 0.82 (0.22, 3.16) | 0.44 (0.08, 2.30) | 0.42 (0.08, 2.21) | 0.21 (0.02, 1.82) | 0.103 |
| Model 3 | 1.00 | 0.94 (0.23, 3.75) | 0.44 (0.08, 2.40) | 0.51 (0.10, 2.79) | 0.22 (0.02, 1.91) | 0.116 |
| PTB |  |  |  |  |  |  |
| Case/N | 12/243 | 10/206 | 8/207 | 10/209 | 6/212 |  |
| Model 1 | 1.00 | 0.98 (0.42, 2.32) | 0.77 (0.31, 1.93) | 0.97 (0.41, 2.29) | 0.56 (0.21, 1.52) | 0.262 |
| Model 2 | 1.00 | 1.09 (0.46, 2.60) | 0.91 (0.36, 2.32) | 1.09 (0.46, 2.60) | 0.63 (0.23, 1.74) | 0.364 |
| Model 3 | 1.00 | 1.07 (0.44, 2.57) | 0.97 (0.38, 2.50) | 1.14 (0.47, 2.78) | 0.62 (0.23, 1.73) | 0.369 |
| Macrosomia |  |  |  |  |  |  |
| Case/N | 7/243 | 5/206 | 5/207 | 3/209 | 11/212 |  |
| Model 1 | 1.00 | 0.84 (0.26, 2.68) | 0.84 (0.26, 2.67) | 0.49 (0.13, 1.92) | 1.85 (0.70, 4.85) | 0.128 |
| Model 2 | 1.00 | 0.88 (0.27, 2.84) | 0.93 (0.29, 3.03) | 0.52 (0.13, 2.07) | 1.98 (0.75, 5.26) | 0.105 |
| Model 3 | 1.00 | 0.92 (0.28, 2.98) | 0.92 (0.28, 3.01) | 0.53 (0.13, 2.09) | 2.00 (0.74, 5.35) | 0.108 |

Model 1: without adjustment. Model 2 adjusted for age, BMI; Model 3 adjusted for the variables in Model 1 plus education background, smoking status, alcohol status, parity, history of miscarriage, nation, history of diabetes, history of hypertension. Abbreviations: PROM: premature rupture of membranes; PTB: preterm birth;
