# Supplementary material for: Association of maternal serum vitamin a levels in the first trimester with the risk of adverse pregnancy outcomes: a prospective cohort study of Chinese women
Source: Front Nutr. 2026 Apr 2;13:1735875. doi: 10.3389/fnut.2026.1735875 (PMC13082939; doi:10.3389/fnut.2026.1735875)
Supplement: Supplementary file 1 [file Supplementary_file_1.zip › Yuan_VitA_AdversePregnancy_Supplementary Figures and Tables/Supplementary Table 2.docx]

Supplementary Material

**Supplementary Table 2.** The correlation of serum vitamin A, Vitamin C and Vitamin E with glucose levels at the first trimester in the cohort study^1^

| Variables | Vitamin A | Vitamin C | Vitamin E |
| --- | --- | --- | --- |
| Vitamin A | 1 |  |  |
| Vitamin C | 0.073^*^ | 1 |  |
| Vitamin E | 0.148^**^ | 0.211^**^ | 1 |
| OGTT Fasting plasma glucose | -0.045 | 0.047 | 0.087^**^ |
| OGTT 1-h blood glucose | -0.084^*^ | -0.008 | 0.011 |
| OGTT 2-h blood glucose | -0.097^**^ | 0.017 | -0.014 |

^1^ Spearman correlation coefﬁcient was shown, * *p* < 0.05，** *p* < 0.01; Abbreviations: OGTT: oral glucose tolerance test.
